# Assessing Matrix Solid-Phase Dispersion Extraction Strategies for Determining Bisphenols and Phthalates in Gilthead Sea Bream Samples

**DOI:** 10.3390/foods13030413

**Published:** 2024-01-27

**Authors:** Dulce L. Soliz, Rosa Ma Garcinuño, Gema Paniagua González, Juan Carlos Bravo, Pilar Fernández Hernando

**Affiliations:** Department of Analytical Science, Faculty of Science, National University of Distance Education, UNED, Las Rozas, 28232 Madrid, Spain; dsoliz@ccia.uned.es (D.L.S.); gpaniagua@ccia.uned.es (G.P.G.); juancarlos.bravo@ccia.uned.es (J.C.B.); pfhernando@ccia.uned.es (P.F.H.)

**Keywords:** plastic contaminants, gilthead seabream, MSPD, bisphenols, phthalates, HPLC-MS

## Abstract

Microplastics (MPs) and nanoplastics (NPs) are widely spread in the environment, generating significant concern due to their potential impact on environmental health. Marine species usually ingest plastic fragments, mistaking them for food. Many toxic compounds, such as plastic additives that are not chemically bound to the plastic matrix, can be released from MPs and NPs and reach humans via the food chain. This paper highlights the development and validation of a straightforward solid–liquid extraction clean-up procedure in combination with a matrix solid-phase dispersion method using high-performance liquid chromatography coupled with mass spectrometry (HPLC-MS) detection, enabling facile, precise, and reliable identification and quantitation of a total of six bisphenols and phthalates in gilthead sea breams. Under the optimized conditions, the developed method showed good linearity (R^2^ > 0.993) for all target compounds. The recoveries obtained were between 70 and 92%. The relative standard deviations (RSDs) for reproducibility (inter-day) and repeatability (intra-day) were less than 9% and 10%, respectively. The limit of detection (LOD) and limit of quantification (LOQ) for the target compounds ranged from 0.11 to 0.68 µg/kg and from 0.37 to 2.28 µg/kg, respectively. A new, efficient extraction methodology for the determination of BPA, BPS, BPF, DBP, DEP, and DHEP in gilthead seabream has been optimized and validated.

## 1. Introduction

Microplastics (MPs) and nanoplastics (NPs) are widely spread in the environment, generating significant concern due to their potential impact on environmental health. In general, it can be said that the MPs/NPs are already part of the food chain by means of mollusks, arthropods, mammals, birds, amphibians, reptiles, fish, etc. These can ingest plastic fragments and/or be entangled in them and drown. They can also be injured or block the digestive organs, reducing ingestion because of a false feeling of fullness, being affected in its energy and nutrition, a low growth rate, blocked enzyme production, decreased fecundity, and, on some occasions, resulting in death [1,2]. Moreover, once plastic particles are inside the animal, they can release their chemical additives, used to improve some properties of plastics and to eliminate or mitigate others that are undesirable [3,4] because these are not chemically bound to the plastic matrix [5].

Bisphenols and phthalates are commonly used as resins, surfactants, and plastic additives and are considered endocrine-disrupting chemicals (EDCs) [6,7]. Bisphenol A (BPA) is the most common bisphenol used as a monomer in the synthesis of polycarbonate, the production of phenol and epoxy resins, polyesters, and polyacrylates, and in the manufacture of food and feed packaging and other plastic materials [8,9].

In an effort to prevent health problems, the use of BPA in the manufacture of materials that may come into contact with food and in production processes is limited in some countries. China established that the value of BPA must be under 0.6 mg/kg in paint, plastics, and adhesives [5], and the European Union has provided a specific migration limit of 0.6 μg/g BPA in food or food simulant [10]. International institutions like the European Food Safety Authority and the US Environmental Protection Agency set a value of 50 μg/kg of body weight/day as an acceptable daily intake of BPA [10]. Nowadays, bisphenol F (BPF) and bisphenol S (BPS) are also used to replace BPA. BPS is used in cleaning products and in phenolic resins, whereas BPF is present in dental sealants, electrical insulating materials, and food packaging and provides durability and increased thickness for coatings in epoxy resins [10,11,12]. However, these compounds have toxicity similar to BPA [5].

Phthalates are commonly used as plasticizers in the manufacture of polyvinyl chloride (PVC) and in other products such as food packaging, glues, toys, adhesives, detergents, etc. In Western Europe, one million tons of phthalates are produced, and about 900,000 tons are used in PVC production [13]. The main drawback is that the phthalates can leach out, mostly with heat and age, because they are bound into the plastics by non-covalent binding [14]. The most commonly used are diethylphthalate (DEP), butylbenzylphthalate (BBP), diisononylphthalate (DINP), and diethylhexylphthalate (DEHP) [15]. Phthalates, DEHP, DBP, BBP, and diisobutyl phthalate (DiBP) have been classified in the European Union (EU) as reproduction toxic substances included in category 1B due to the danger presented, that is, these compounds can be used only with prior authorization from the EU [16]. In China, the quality limits for food and food additives are set at 0.3 mg/kg for DBP and 1.5 mg/kg for DEHP [17].

There is growing concern regarding the ingestion of microplastics by fish and the potential effects on both marine ecosystems and human health, given the integral role fish play in the human diet and marine food webs. The gilthead seabream (*Sparus aurata*) emerges as a particularly relevant species in this context, being one of the most widely consumed fish species by humans. Notably, it holds the distinction of being the third most produced fish species in Europe and is the primary species cultivated in the Mediterranean region. Statistics from reputable sources such as FAO, FEAP, and APROMAR indicate that the total aquaculture production of gilthead seabream in Europe and the rest of the Mediterranean in 2020 reached a substantial 278,199 tons. This underscores the species’ economic and dietary significance in the region. Furthermore, the gilthead seabream is recognized as a predator in the wildlife hierarchy, adding to its relevance in studies and research concerning the bioaccumulation of microplastics (MPs) and other contaminants derived from plastic [18]. Given its ecological and economic importance, the gilthead seabream serves as a focal point for understanding the potential impacts of plastic pollution in aquatic environments and assessing the associated risks to both marine ecosystems and human consumers.

Currently, the occurrence of bisphenols (BPs) and phthalates (PHs) in gilthead seabream has not been studied in detail, despite the implications that the consumption of this fish contaminated with these compounds could have for human health. To our knowledge, there has not been published in the literature a comprehensive study that addresses the extraction and quantification of these compounds in sea bream samples. At present, the detection and analysis of these contaminants in sea food are carried out by sensitive analytical techniques, like high-performance liquid chromatography (HPLC), coupled with both ultraviolet (UV) and fluorescence detection (FD) [19], gas chromatography with mass spectrometry (GC–MS) [20] and tandem mass spectrometry (GC–MS/MS) [21], and liquid chromatography with mass spectrometry (LC-MS) or tandem mass spectrometry (LC-MS/MS) [22]. High-resolution mass spectrometry (HRMS), time-of-flight mass spectrometry (TOFMS) [23], or orbitrap mass analyzers [24] are also used. However, successful analysis can only be achieved with a suitable sample preparation procedure. In this way, techniques such as DLLME (dispersive liquid–liquid microextraction), QuEChERS (quick, easy, cheap, effective, rugged, and safe), d-SPE (dispersive solid-phase extraction), solvent extraction (SE), and solid-phase microextraction (SPME) have proven to be key factors in optimizing analyte extraction and reducing matrix effects [25,26].

A sample preparation method based on the combination of QuEChERS and DLLME procedures has been employed for the determination of bisphenol in mussels and fish muscle samples [20] and in milk samples using HPLC–FLD detection [27,28]. It proposed the determination of a mixture of bisphenols and derivatives in canned beverages, such as tea, cola, tonic, beer, and water, and in canned food, such as lentils, meatballs, chickpeas, and sweet corn, by a supramolecular-based comprehensive sample treatment platform and LC-MS/MS.

Hidalgo-Serrano et al. (2020) [29] developed the simultaneous determination of several phthalates in seafood species by applying a pressure liquid extraction (PLE) method and liquid chromatography coupled to high-resolution mass spectrometry (LC-HRMS).

Matrix solid-phase dispersion (MSPD) has gained widespread acceptance as a highly efficient alternative to traditional methodologies for isolating organic chemicals from diverse and complex matrices. MSPD distinguishes itself by offering a simultaneous extraction and purification process, utilizing a minimal amount of sample and solvent. This method is recognized for its speed, cost-effectiveness, and environmental friendliness [30]. The inherent advantages of MSPD lie in its ability to streamline the extraction and purification steps, contributing to enhanced efficiency and reduced resource utilization. By employing a smaller quantity of sample and solvent, MSPD aligns with the principles of sustainability and cost-effectiveness. Its environmentally friendly attributes further underscore its appeal in analytical chemistry, making it a convenient technique for the analysis of complex sample matrices.

Generally, the methods developed to date are protein-rich matrices with lower fat content. In this study, a species (gilthead seabream) whose total lipid content is significant has been chosen [31].

**Figure 1:**
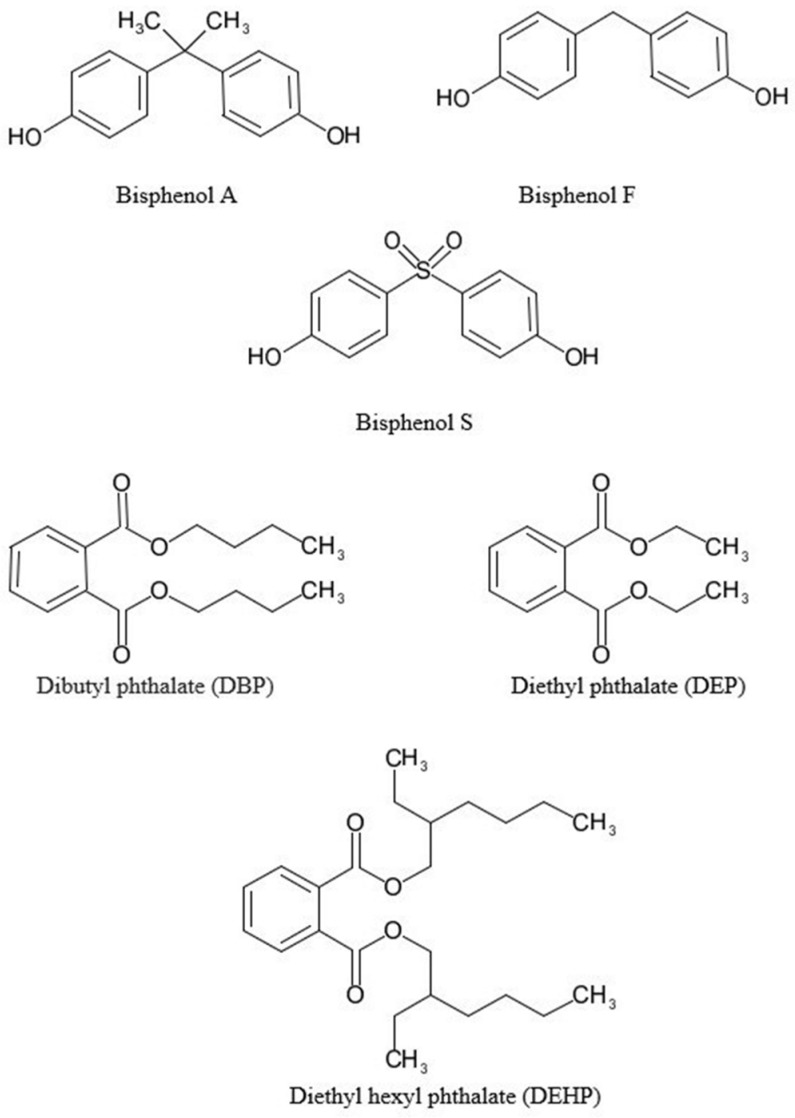
Chemical structures of bisphenol A (BPA), bisphenol F (BPF), bisphenol S (BPS), dibutyl phthalate (DBP), diethyl phthlate (DEP), and diethyl hexyl phthalate (DEHP).

For these reasons, the present study aims to develop a new fat extraction-MSPD procedure combined with HPLC-MS for the determination of bisphenols (BPA, BPS, BPF) and phthalates (DBP, DEP, DHEP) (see Figure 1 for chemical structures) from *Sparus aurata* (gilthead seabream). Parameters involved in MSPD extraction, such as ratio sorbent/sample, type of sorbent, and type and amount of solvent extraction, were optimized. The method was validated using gilthead seabreams.

## 2. Materials and Methods

### 2.1. Reagents, Standards, and Materials

Methanol (MeOH), acetonitrile (MeCN), chloroform, dichloromethane, petroleum ether, and diethyl ether (HPLC-grade purity) were obtained from Macron Fine Chemicals (Barcelona, Spain), and acetic acid (99.9% purity) from J.T. Baker (Madrid, Spain). The analytical standards of bisphenol S (BPS, purity ≥ 98%), bisphenol F (BPF, purity ≥ 98%), bisphenol A (BPA, purity ≥ 99.9%), di(2-ethylhexyl) phthalate (DEHP, purity ≥ 99.5%), dibutyl phthalate (DBP, purity ≥ 99%), and diethyl phthalate (DEP, purity ≥ 99%) were obtained from Sigma Aldrich (Madrid, Spain). Individual standard stock solutions of the selected compounds were prepared in methanol and maintained in darkness at 4 °C until use. A concentration of 1000 mg/L was prepared for BPA, BPS, and phthalates (DEHP, DEP, and DBP), and a concentration of 100 mg/L for BPF. Daily working standard solutions were prepared by appropriate dilution with the mixture MeCN/water (85:15, *v*/*v*). A Milli-Q water system (Merck Millipore, Madrid, Spain) was used to obtain ultrapure water (18 MΩ/cm, 25 °C).

Florisil (60–100 mesh) from Acros Organics (Madrid, Spain), sodium sulfate anhydrous (Na_2_SO_4_, purity ≥ 99.9%), and washed sea sand (0.25–0.30 mm) from Panreac (Barcelona, Spain) were employed as sorbents for the sample extraction method. The silanized glass wool used was purchased by Panreac (Barcelona, Spain). Glass SPE cartridges (6 mL) used for packing the sorbent material were obtained from J.T. Baker (Deventer, The Netherlands). Evaporator/concentrator equipment (TECHNE, Long Branch, NJ, USA) was used to evaporate the samples.

### 2.2. Samples

Fresh gilthead seabreams (*Sparus aurata*) obtained from aquaculture were acquired from a local supermarket in Madrid, Spain. Meticulous sample preparation involved precise dissection of each sample, accomplished by delicately opening the abdominal cavity with a sterile knife. This process allowed the separation of different components, including the head, skin, viscera, central bone, and fillets. To ensure the representativeness of the samples, a careful approach was taken by homogenizing a notable portion of the fillets using a high-quality blender. The homogenized samples were then diligently preserved at −20 °C, maintaining their freshness and integrity for further analysis. This methodology allows us to obtain precise and reliable results in our study.

### 2.3. Sample Extraction Procedure

**Figure 2:**
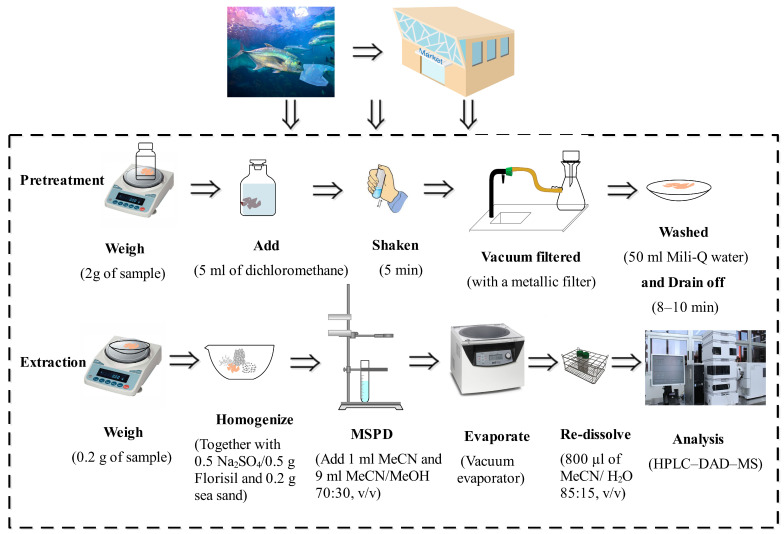
Optimized sample extraction procedure.

A representative portion of homogenized sea bream muscle was accurately weighed (2 g) and transferred to a 15 mL glass tube. Then, a volume of 5 mL of dichloromethane was added. The mixture was shaken for 5 min and vacuum-filtered using a metallic filter. After washing with a volume of 50 mL of Mili-Q water and draining off for 8–10 min, the sample of fat content was submitted to an already optimized MSPD treatment [32] (Cañadas et al., 2021). For this, a portion of 0.2 g of the clean sample was blended with 0.5 g of Florisil (used as a disrupting agent, 60–100 mesh), 0.2 g of washed sea, and 0.5 g of N_2_SO_4_ (anhydrous component, purity ≥ 99.9%) for approximately 10 min, employing a glass mortar and pestle to perform a full disruption and dispersion of the sample on the solid supports. The homogeneous and dry material was packed into a SPE glass cartridge. A piece of glass wool was placed at the bottom of the SPE tube to prevent the sample from leaking out, and a slight amount of Na_2_SO_4_ was deposited on top of the mixture. It is necessary to add the solid mixture into the MSPD column in several portions to avoid air pockets and ensure homogeneous packing. The target compounds were properly eluted dropwise by gravity from the SPE column previously conditioned using 1 mL of acetonitrile by three static extraction steps (5 min) and 9 mL of methanol/acetonitrile (30:70, *v*/*v*). The collected extracts were evaporated to dryness under a nitrogen flow at room temperature, and the residue was reconstituted in 800 µL of MeCN/H_2_O (85:15, *v*/*v*). The optimized procedures depicted in Figure 2.

### 2.4. Chromatographic Analysis

Chromatographic separations were carried out using a column ACE-1210-1546 C18-PFP (150 × 4.6 mm) obtained from Symta (Madrid, Spain). Ultrapure water Milli-Q (component A) and acetonitrile (component B), both containing 0.1% of acetic acid, were used as the mobile phase. Gradient elution started at 45% B, linearly increased to 80% B in 30 min, 100% B in 1 min, and maintained 100% B for 9 min. Subsequently, the column was reequilibrated at the starting conditions for 10 min. An injection volume of 40 μL was used. Analytes were separated at 0.8 mL min^−1^ operating at 20 °C.

The HPLC system (Agilent 1260 series) was supplied by Agilent Technologies. It consisted of an autosampler, a quaternary pump, a thermostatted column compartment, a diode array spectrophotometric detector (DAD), and a 6100 simple quadrupole mass spectrometer with an electrospray ionization (ESI) interface (Agilent) controlled by ChemStation (Rev.B.04.02) software. To optimize sensitivity, quantitative assessments of peak areas were meticulously conducted by selecting the most suitable detection wavelength for each compound. The wavelength values were judiciously determined based on previous research conducted by our group [32]. Analytes were quantified by external calibration, with the peak area of chromatographic peaks at 210 nm as the optimum wavelength for all of them.

The optimum MS parameters were as follows: positive and negative ionization modes for phthalates and bisphenols, respectively; temperature of the capillary was set at 350 °C; capillary voltage used was 5000 V; nebulizer pressure was 60 psi, gain 2; and sheath N_2_ flow was 11 L min^−1^_._ The optimized parameters are summarized in Tables S1 and S2.

### 2.5. Quality Assurance

In our commitment to maintaining the highest standards of precision and reliability, stringent control measures were meticulously integrated into both laboratory and sampling procedures. This was paramount to prevent any background contamination and to ensure an accurate estimation of analyte concentrations. To achieve this, a comprehensive cleaning regimen was implemented for all glassware and dissection materials. Prior to use, each item underwent a meticulous process involving washing with dishwashing liquid, thorough rinsing with Milli-Q water, and a final rinse with acetone after drying. This rigorous cleaning protocol aimed to eliminate any potential sources of contamination.

Additionally, the use of plastic labware was strictly avoided throughout the sampling, sample treatment, and analysis phases. This deliberate decision was made to mitigate the risk of introducing contaminants from plastic materials into the samples. Furthermore, to validate the precision of our measurements, analytical blanks were systematically carried out at every step of the procedure. These blanks, specific to each stage, served as a critical control mechanism, ensuring the accuracy and reliability of the entire analytical process—from sample collection through treatment to final analysis.

## 3. Results and Discussion

### 3.1. Sample Extraction Procedure

The proposed extraction procedure for BPs and PHs determination was based on previous research reported by the authors [32], in which a MSPD methodology was used. Briefly, spiked samples at adequate analyte concentration are disrupted with a mixture of sorbents (Florisil, anhydrous sodium sulfate, washed sea sand, 5:5:2) and packed onto a SPE column. Then, after conditioning the column, the compounds were eluted using 9 mL of methanol/acetonitrile (30:70, *v*/*v*). The obtained extracts were evaporated to dryness, reconstituted in 400 µL of a mixture of MeOH/H_2_O (85:15, *v*/*v*), and injected onto HPLC-MS. However, the application of this same extraction method to the seabream samples has not provided the expected results since the extracts were not cleaned enough to be injected into the HPLC. Then, an additional clean-up step was required to remove interferences such as lipids and proteins due to the sample complexity. The first experiments aimed at cleaning the extracts obtained from the MSDP. For this purpose, SPE cartridges with different sorbents, such as active carbon, alumina, and C-18, were tested. MSPD extracts were loaded onto clean-up SPE cartridges after being conditioned, and target analytes were eluted using a methanol/acetonitrile mixture. These purification sorbents provided yield recoveries lower than 30% for most of the analytes, and when active carbon was used, BPF and DHEP were not eluted from the column. To improve the extraction procedure, a previous saponification step was tested before the MSPD protocol.

For that, 2 mL of 0.01 M NaOH were loading onto the MSPD column. The extract containing interferences material was discarded and the column was submitted to MSPD extraction. Recoveries of some of the compounds increased slightly, reaching values between 15 and 65% and BPF could not be eluted from the column.

In response to the unsatisfactory extraction yield, a deliberate shift in focus was undertaken for subsequent experiments, aiming to enhance the cleaning of the sample before initiating the Matrix Solid-Phase Dispersion (MSPD) protocol. A diverse range of compounds, including NaOH at 0.5 M, hexane, dichloromethane, petroleum ether, and diethyl ether, were systematically evaluated for their efficacy in improving the extraction efficiency. Recognizing the significance of this optimization, approximately 2 g of the sample was carefully chosen and subjected to a meticulous solid–liquid extraction to eliminate potential interferences.

To execute these experiments, a precisely measured volume of 5 mL of an appropriate cleaning solvent was introduced to the sample. Employing a robust procedure, the mixture underwent vigorous shaking for a duration of 10 min, followed by centrifugation for 5 min at 4000 rpm. The resulting supernatant, containing undesired interferences, was meticulously collected and discarded. Subsequently, the solid fraction was carefully drained off and reserved for the subsequent MSPD protocol.

This approach, targeting sample cleaning and pre-treatment, marks a strategic point in the experimental design. These adjustments will not only rectify the challenges encountered in the initial extraction process but also contribute to the overall optimization and effectiveness of the analytical methodology. The comprehensive results of these innovative experiments are thoughtfully documented and presented in detail in Table 1.

**Figure 3:**
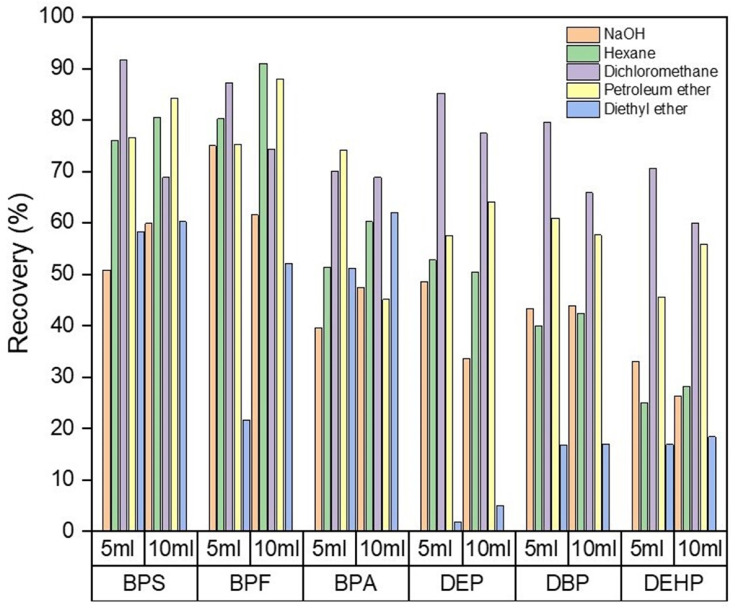
Analyte recoveries using two consecutive cleaning processes using 5 mL of different cleaning agents before MSPD extraction.

Dichloromethane provided the best results, obtaining recoveries ranging between 70 and 92% for all the compounds. To optimize the previous cleaning step, different volumes of solvents were tested (5–10 mL). It was observed that volumes higher than 5 mL did not improve analyte recovery in any case. The efficiency of the cleaning procedure was also checked by carrying out two consecutive processes using 5 mL of cleaning agent. Figure 3 compares analyte recoveries obtained by a single extraction of 5 mL and two consecutive clean-up processes of 5 mL each for all the agents tested. It can be observed that no significant differences were obtained when an additional clean-up step was performed; thus, only one clean-up step was used. Then, a prior sample clean-up using 5 mL of dichloromethane was considered optimal for the validation of the method. All assays were carried out in duplicate using matched samples, and blank assays were performed for all the experiments.

**Figure 4:**
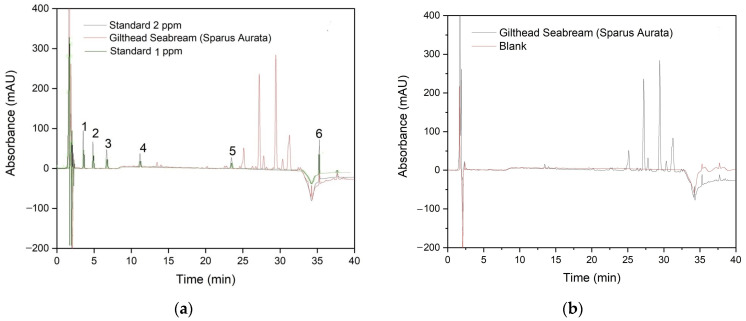
(**a**) Chromatograms at 210 nm of a standard sample of contaminants derived from plastics at different concentrations (__ 2 ppm/__ 1 ppm) and gilthead seabream sample (__). Peaks: (1) BPS, (2) BPF, (3) BPA, (4) DEP, (5) DBP, and (6) DEHP. (**b**) Chromatograms at 210 nm of a gilthead seabream sample (__) and the blank (__).

Figure 4a shows the chromatogram corresponding to a standard sample at different concentration levels and a chromatogram of a gilthead seabream sample after sample extraction. The presence of interfering substances can be observed; however, it is possible to quantify the compounds of interest. Figure 4b includes a comparison between a chromatogram of the gilthead seabream sample and a blank chromatogram.

### 3.2. Validation of the MSPD Method

The developed methodology was validated by the evaluation of the following parameters: linearity range, accuracy, precision (repeatability and reproducibility), limits of detection (LODs), and limits of quantification (LOQs) using spiked gilthead seabream samples (Table 2).

The linearity range was evaluated by constructing matrix-matched calibration curves at concentration levels of each analyte ranging between 0.5 and 50 µg/kg and plotting peak area versus concentration. The determination coefficients (R^2^), calculated by the least squares regression model (0.993–0.999), indicated high linearity, and the quantification of the analytes is adequate in the tested concentration range.

The accuracy was assessed by testing fish samples spiked at three concentration levels at different sections of the linearity range: 0.5 μg/kg (low), 5 μg/kg (medium), and 10 μg/kg (high) sections of the linearity range (0.5–50 μg/kg). The samples were analyzed in quintuplicate using the developed procedure. The recoveries obtained ranged between 70 and 93%, with relative standard deviation (RSD) values ranging 3–10% for all the analytes and at all concentration levels tested.

The precision of the procedure was calculated by analyzing spiked samples at three concentration levels, considering intra-day (repeatability) and inter-day (reproducibility) assays (n = 5). The inter-day and intra-day variability, evaluated as % of RSD, was in the range of 3–9% and 4–10%, respectively, for all the analytes.

The detection (LOD) and quantification limits (LOQ) were established following the indications of the FDA Guidance for Industry [33]. The LODs of the analytes ranged from 0.11–0.68 µg/kg, while their LOQs were between 0.37 and 2.28 µg/kg. These data were comparable with results from food samples published in the literature [21,34].

## 4. Conclusions

A pioneering and highly efficient extraction methodology has been meticulously developed to quantify three bisphenols (BPA, BPS, and BPF) and three phthalates (DBP, DEP, and DHEP) in gilthead seabreams. This innovative approach involved the optimization and validation of a dual extraction protocol, incorporating a dichloromethane cleaning step and a matrix solid-phase dispersion (MSPD) technique, culminating in high-performance liquid chromatography–mass spectrometry (HPLC-MS) detection. The method exhibited good performance, generating clean extracts with adequate recovery rates ranging between 70 and 92% for all targeted analytes. The relative standard deviations (RSD) fell within the narrow range of 3–10%, attesting to the method’s precision and reliability. This thorough sample treatment protocol not only ensured the removal of matrix interferences in seafood but also demonstrated its versatility. The limits of detection (LOD) and limits of quantification (LOQ) achieved for all compounds were consistently lower than 0.68 and 2.28 µg/kg, respectively. Such sensitivity positions the methodology as an adept tool for the meticulous monitoring of contaminated samples. The validated analytical method was successfully applied to determine the specified bisphenols and phthalates in gilthead seabream samples, showcasing its potential for routine analysis. Significantly, and to the best of our knowledge, a noticeable void exists in the published literature regarding the simultaneous determination of bisphenols and phthalates in seabream samples. Thus, the developed methodology not only fills this research gap but also stands as a novel and impactful contribution to the field, emphasizing its relevance in advancing analytical techniques for seafood analysis.

## Figures and Tables

**Table 1:** Analyte recoveries using 5 mL of different cleaning agents before MSPD extraction.

Cleaning Solvent	% Recovery					
	BPS	BPF	BPA	DEP	DBP	DHEP
NaOH 0.5 M	50.81	74.96	39.60	48.52	43.28	33.05
Hexane	76.05	80.24	51.34	52.75	39.93	24.92
Dichloromethane	91.77	87.23	70.06	85.23	79.54	70.55
Petroleum ether	76.58	75.26	74.22	57.53	60.85	45.59
Diethyl ether	58.32	21.62	51.10	1.860	16.77	16.90

**Table 2:** Analytical characteristics of BPs and DPs in seabream samples (n = 5).

Analyte	Linearity		Spiking Level µg/kg	Recovery ± RSD %		LOD µg/kg	LOQ µg/kg
	Concentration Range µg/kg	R^2^		Inter-Day	Intra-Day		
BPS	0.5–50	0.999	0.50	92.3 ± 6.21	90.5 ± 7.57	0.23	0.78
			5.00	91.9 ± 5.03	92.5 ± 5.82		
			10.0	92.0 ± 5.30	91.8 ± 5.92		
BPF	0.5–50	0.999	0.50	86.3 ± 7.22	84.9 ± 8.59	0.11	0.37
			5.00	87.6 ± 6.75	88.2 ± 6.81		
			10.0	83.4 ± 6.27	85.7 ± 6.94		
BPA	0.5–50	0.994	0.50	70.2 ± 4.50	69.9 ± 4.85	0.27	0.89
			5.00	71.5 ± 4.14	70.9 ± 4.62		
			10.0	70.9 ± 3.92	71.2 ± 4.50		
DEP	0.5–50	0.996	0.50	85.4 ± 3.87	81.3 ± 4.67	0.32	1.10
			5.00	86.5 ± 3.05	87.7 ± 3.91		
			10.0	86.3 ± 3.19	88.5 ± 4.10		
DBP	0.5–50	0.993	0.50	79.6 ± 7.14	77.5 ± 8.01	0.21	0.69
			5.00	78.2 ± 7.22	78.9 ± 7.97		
			10.0	79.1 ± 6.51	78.3 ± 7.15		
DHEP	0.5–50	0.996	0.50	71.1 ± 9.04	70.1 ± 10.0	0.68	2.28
			5.00	72.4 ± 8.21	71.5 ± 9.54		
			10.0	70.2 ± 8.08	73.5 ± 8.21		

## Data Availability

The original contributions presented in the study are included in the article/Supplementary Material, further inquiries can be directed to the corresponding author.

## Supplementary Materials

The following supporting information can be downloaded at: https://www.mdpi.com/article/10.3390/foods13030413/s1, Table S1: Target compounds, ions, cone voltage, and retention time in ionized positive (ESI+) mode for HPLC–MS detection; Table S2: Target compounds, ions, cone voltage, and retention time in ionized negative (ESI−) mode for HPLC–MS detection.
