# Supplementary material for: Assessing Matrix Solid-Phase Dispersion Extraction Strategies for Determining Bisphenols and Phthalates in Gilthead Sea Bream Samples
Source: Foods. 2024 Jan 27;13(3):413. doi: 10.3390/foods13030413 (PMC10855760; doi:10.3390/foods13030413)
Supplement: Supplementary file 1 [file foods-13-00413-s001.zip › foods-2794452-supplementary.pdf]

Supporting information

Article

Assessing Matrix Solid-Phase Dispersion Extraction Strategies for Determining Bisphenols and Phthalates in Gilthead Sea Bream Samples

Dulce L. Soliz †, Rosa Ma Garcinuno *, Gema Paniagua González, Juan Carlos Bravo and Pilar Fernández Hernando

Department of Analytical Science, Faculty of Science, National University of Distance Education, UNED, Las Rozas, 28232 Madrid, Spain; dsoliz@ccia.uned.es (D.L.S.); gpaniagua@ccia.uned.es (G.P.G.); juancarlos.bravo@ccia.uned.es (J.C.B.); pfhernando@ccia.uned.es (P.F.H.)

* Correspondence: Correspondence: rmgarcinuno@ccia.uned.es

† Researcher in training at the International Doctoral School of UNED, in the Doctoral Program in Sciences.

Table of contents

Table S1 Target compounds, ions, cone voltage and retention time in ionized positive (ESI+) mode for HPLC–MS detection.

Table S2 Target compounds, ions, cone voltage and retention time in ionized negative (ESI−) mode for HPLC–MS detection.

Table S1. Target compounds, ions, cone voltage and retention time in ionized positive (ESI+) mode for HPLC–MS detection.

Analytes	Ion SIM	Cone Voltage, eV	Retention time (t_R), min
DEP	149, 177	100	10.489
DBP	149, 205	100	22.679
DEHP	149, 167, 279	120	34.976

Table S2. Target compounds, ions, cone voltage and retention time in ionized negative (ESI−) mode for HPLC–MS detection.

Analytes	Ion SIM	Cone Voltage, eV	Retention time (t_R), min
BPS	92, 108, 156, 249	120	3.376
BPF	98, 183, 199.1, 200	120	4.563
BPA	119, 213, 228	120	6.244
